# Staged urethroplasty with groin full-thickness skin graft for managing complex anterior urethral strictures: surgical outcomes and predictive factors

**DOI:** 10.1007/s00345-024-05049-3

**Published:** 2024-05-22

**Authors:** Min Chul Cho, Jooho Lee, Soo Woong Kim

**Affiliations:** 1 Department of Urology, Seoul National University College of Medicine and Seoul National University Boramae Medical Center, Seoul, 07061 Korea; 2 Seoul National University Hospital, Seoul, 03080 Korea; 3 Department of Urology, Seoul National University College of Medicine and Seoul National University Hospital, 101 Daehak-Ro, Jongno-Gu, Seoul, 03080 Korea

**Keywords:** Urethral stricture, Staged urethroplasty, Full thickness skin graft, Groin

## Abstract

**Purpose:**

To describe outcomes of staged-urethroplasty in complex anterior urethral strictures using full-thickness-skin-graft (FTSG) harvested from the hairless groin area, and to identify factors influencing successful outcomes.

**Methods:**

Through retrospective chart review, we identified a total of 67 men who underwent the first-stage operation (grafting) using groin-FTSG for staged-urethroplasty to treat complex anterior urethral strictures unsuitable for one-stage urethroplasty. Among these, 59 underwent the second-stage operation (tubularization) at a median duration of 5.1-months after grafting. Patients were assessed for outcomes as scheduled after tubularization outcomes were analyzed only for 48 patients for whom ≥ 1-year follow-up data after tubularization were available. Their mean follow-up duration was 27.1 months. Success was defined as achieving physiologic voiding without requiring further procedures.

**Results:**

Median stricture-length was 5.5 cm in all 67 patients. After grafting, neourethral-opening-narrowing occurred in 18. Partial graft-loss occurred in 8, of whom only 3 underwent re-grafting. The percentage of patients who achieved successful outcomes was 81.3%. Improvements in maximum-urine-flow-rate and post-void-residual-urine-volume were maintained until the last follow-up visit. A urethrocutaneous-fistula occurred in one patient, while meatal-stenosis occurred in two. On multivariate-regression-analysis, the presence of neourethral-opening-narrowing was the only predictor of non-success after tubularization. Furthermore, the presence of hypertension, longer stricture-length, and a history of prior direct-vision-internal-urethrotomy were predictors of the occurrence of neourethral-opening-narrowing.

**Conclusion:**

Staged-urethroplasty using groin-FTSG is well worth considering as a useful therapeutic option for complex anterior urethral strictures, with an acceptable success rate and low morbidity. The absence of neourethral-opening-narrowing after the first-stage operation leads to success.

**Supplementary Information:**

The online version contains supplementary material available at 10.1007/s00345-024-05049-3.

## Introduction

Treating complex anterior urethral stricture remains one of the most challenging issues for urologists. To reduce morbidities related to multiple procedures, there has been a recent expansion of indications for one-stage urethroplasty to treat complex anterior urethral strictures [1]. However, the necessity of staged urethroplasty still remains for treating complex anterior urethral strictures unsuitable for one-stage urethroplasty [1–4].

For the past decades, several sources of graft materials have been introduced for staged urethroplasty [1, 5]. Among these, buccal-mucosa graft (BMG) has been widely applied in substitution urethroplasties, because it has some advantages including compatibility with a wet environment [5–7]. However, BMG has some drawbacks such as donor-site morbidity and a limited quantity of available tissue [8, 9]. Meanwhile, several sources of extragenital full-thickness skin grafts (FTSGs), including postauricular skin and abdominal-FTSG, have played roles in staged urethroplasty [1]. However, current evidence suggests their own shortcomings, such as a high revision rate, donor-site morbidity including keloid formation, cosmetic issues, delayed hair growth and more stricture recurrence compared to other sources of graft material [1, 10–13].

The hairless groin area offers a good source of skin for grafting due to its relatively ample size, high elasticity, high graft survival rate and minimal morbidity at the donor site, as reported by a previous study about reconstructive maxillofacial surgery [14, 15]. Furthermore, the resulting scar in a hairless groin area can be concealed by the inguinal crease after the primary closure of the harvested wound [14]. Therefore, hairless groin skin can be a good source of extragenital-FTSG for staged urethroplasty in treating complex anterior urethral stricture. Our study, for the first time, aimed to describe surgical outcomes of staged urethroplasty in complex anterior urethral stricture using hairless groin-FTSG and to identify factors influencing stricture recurrence.

## Methods

### Study design

This study was approved by our Institutional-Review-Board. All data were collected form retrospective chart review. We identified a total of 67 men with complex anterior urethral stricture who underwent grafting using hairless groin-FTSG for staged urethroplasty between 2003 and 2022. Among these, a total of 59 patients underwent the second-stage operation (tubularization) at a median duration of 5.1-months (range: 4.0–32.0) after the first-stage operation.

The preoperative evaluations included medical/surgical history, physical examination, urinalysis, blood tests, uroflowmetry, and retrograde urethrography. A voiding-cystourethrography or urethroscopy was performed if indicated. Staged urethroplasties were performed in 32 patients who had experienced failed hypospadias repair or prior urethroplasty. Additional indications included 35 patients with very poor urethral plates for reconstruction due to multiple prior dilations or direct-vision-internal-urethrotomies (DVIU).

### Surgical techniques: the first-stage (grafting)

The most distal part of the urethral stricture was identified using a bougie. A guidewire or a 6Fr-ureteral catheter was placed through the urethral lumen to avoid the risk of losing the proximal urethral lumen during surgery if the urethral stricture was severe. The ventral surface of urethra was opened longitudinally, and the incision was extended to 1-cm proximal and distal to the stricture tract until at least a 24Fr-bougie could pass. In general, the urethral plate was preserved if possible, except when it was unusable (< 0.5 cm wide due to significant spongiofibrosis). Panendoscopy was performed to inspect the urethra proximal to the stricture point. Additional surgical corrections were made to the proximal urethra if needed.

**Fig. 1:**
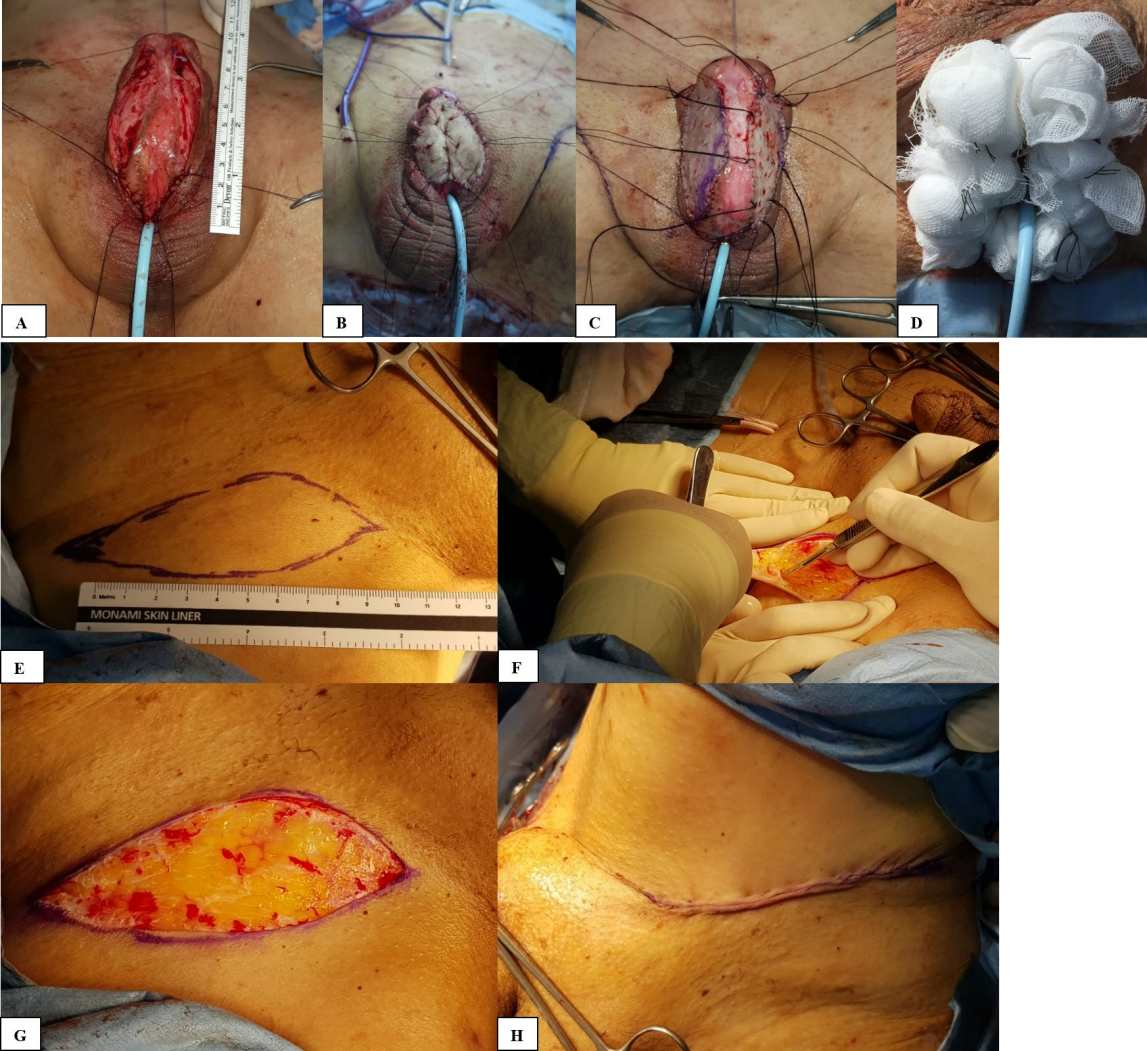
The first-stage operation (grafting). **A** Measuring the extent of urethral defect after excising nonviable tissues. **B** The graft was sutured as a replacement of the urethral plate. **C** The graft was sutured between skin edges and the urethral plate and quilted on the underlying corpus cavernosum. **D** Bolster dressing **E** Graft outlining at groin area **F** Graft is sharply excised with a scalpel. **G** Exposed dermal defect **H** The donor site is repaired with subcuticular suture. Informed consent for publication of the images was obtained from a patient

The extent of the urethrotomy was measured to prepare for graft harvest (Fig. 1A). We intended to achieve a 3-cm-wide native urethral plate and graft bed along the whole length of the reconstruction to provide an approximately 24Fr-lumen for tubularization. The FTSG was harvested by plastic surgeons from a hairless groin area with available loose adjacent skin to achieve primary closure (Fig. 1E–H). After the graft was designed elliptically and excised with a scalpel without elevating the underlying tissue, graft defatting was performed. The harvested graft was approximately 10% longer than the measured length of the urethral defect to achieve a large enough plate. If some viable plate remained, the graft was divided and sutured between skin edges and the urethral plate bilaterally using 5-0 polyglactin suture. If no plate was viable, the graft was sutured as a replacement of the urethral plate (Fig. 1B). Quilting suture with 5-0 rapid-polyglactin was performed to affix the graft to the underlying corpus cavernosum (Fig. 1C). A 14Fr-Foley catheter was indwelled. Bactigras gauze and a bolster-dressing of moisturized artificial cotton were placed followed by tie-over suture for 5- to 7-days postoperatively (Fig. 1D). The timing for catheter removal was determined based on the status of graft take. Patients underwent uroflowmetry, and No.7 Nelaton tubes were inserted through the neourethral-opening at 1-week, 4-weeks, and 2- to 3-months after the removal of the Foley catheter. If the tube passed easily through the neourethral-opening, the second-stage operation was planned for 4- to 6-months after the first-stage operation. If the tubes did not pass easily, the neourethral-opening was dilated using urethral sounds. If the neourethral-opening narrowing persisted after the urethral dilation, we planned revision surgery such as Heineke-Mikulicz-strictureplasty for neourethral-opening narrowing during the second-stage operation. Neourethral-opening narrowing was defined as having narrow lumen unable to pass No.7 (14 Fr) Nelaton tube through neourethral-opening.

### Surgical techniques: the second-stage (tubularization)

The diameter of the neourethral-opening was assessed using a bougie. If the neourethral-opening was narrow (< 20Fr with bougie), Heineke-Mikulicz-strictureplasty was performed during tubularization. Regarding the Heineke-Mikulicz-strictureplasty, a longitudinal urethrotomy was made using a scalpel over the narrow neourethral-opening on its dorsal side, and then it was closed transversely with one-layer interrupted sutures. The graft was mobilized and tubularized over a 20Fr-Foley catheter with 5-0 polyglactin subcuticular continuous suture. Dartos was mobilized to cover the suture line with 5-0 polyglactin interrupted suture. A 14Fr-Foley catheter was indwelled.

### Postoperative follow-up and definition of outcomes

Patients were assessed for micturition symptoms or complications and underwent uroflowmetry at 1-week, 1-, 3-, 6-, 9-, and 12-months after the tubularization, and then annually thereafter. Patients who experienced aggravated voiding symptoms, increased post-void residual urine volume (PVR), or had a maximum urinary-flow-rate (Qmax) < 10 mL/s even without difficulty voiding, underwent cystourethroscopy and urethral calibration by sound dilation to identify the strictures. Success was defined as (1) physiologic voiding, and (2) no need for additional procedures.

### Statistical analysis

The variables (Qmax and PVR) were evaluated for statistical differences between the baseline measures and the measures obtained after the surgery using the Wilcoxon signed-ranks test for continuous variables. Comparative analyses between the successful and non-successful groups or between patients with neourethral-opening narrowing and without neourethral-opening narrowing were performed using the Mann–Whitney U test for continuous variables and the chi-square test or Fisher’s exact test for categorical variables. The potential predictor variables were included in the univariate regression model to identify factors influencing successful outcomes. Variables with a p-value < 0.05 on univariate analyses (the presence of hypertension and the presence of neourethral-opening narrowing) were included in multivariate analyses. Statistical-significance was set at a 5%-level. The Statistical-Package-for-the-Social-Sciences (Version 22.0. Armonk, NY: IBM-Corp.) was used.

## Results

### Baseline characteristics and perioperative data of the first-stage operation in all 67 patients (Table 1S in supplementary material)

The median stricture length was 5.5 cm (range: 1.0–12.0). After grafting, neourethral-opening narrowing occurred in 18 cases (26.9%). The narrowing occurred only in the proximal neourethral-opening in 13, only in the distal neourethral-opening in 2, and in both the proximal and distal neourethral-openings in 3. Partial graft loss occurred in 8 patients, of whom only 3 underwent re-grafting.

### Perioperative data and follow-up outcomes of the second-stage operation in 48 eligible patients

A total of 8 patients chose not to undergo tubularization as they were satisfied with the improved voiding following the first-stage operation. The successful outcomes were analyzed only for 48 patients for whom ≥ 1-year follow-up data after tubularization were available. The mean (± SD) follow-up duration in these 48 patients was 27.1 months (± 14.1). A successful outcome was observed in 39 (81.3%). The improvements in Qmax and PVR were maintained until the last follow-up visit (Fig. 2S in supplementary material).

In the non-successful group (n = 9), the median time to stricture recurrence was 4.5 months (range: 0.7–27.8). Among the six patients in whom neourethral-opening narrowing occurred after grafting in the non-successful group, two patients underwent repeat staged urethroplasty but continued to have an unsuccessful outcome and undergo periodic urethral dilation or self-catheterization. Among the remaining four patients, one reported satisfaction with self-catheterization, while the other two patients continued to undergo periodic urethral dilation or self-catheterization. Among the three patients in whom neourethral-opening narrowing did not occur after grafting in the non-successful group, one achieved a satisfactory outcome after undergoing redo staged urethroplasty, and another had a satisfactory outcome after a single instance of urethral dilation.

### Complications after the second-stage operation

In terms of complications, one patient initially experienced a urethrocutaneous fistula, which later closed spontaneously. Furthermore, two patients had meatal stenosis, which was managed through urethral dilation. Additionally, 17 patients reported post-micturition dribble, while 5 patients complained of spraying.

### Predictive factors of surgical outcomes

The non-successful group had a higher prevalence of hypertension and a higher rate of neourethral-opening narrowing after grafting, compared to the successful group (Table 2S in supplementary material). On multivariate logistic-regression analysis, the presence of neourethral-opening narrowing was the only independent predictor of a non-successful outcome after tubularization (adjusted odds ratio [95% confidence-interval]: 5.733 [1.102–27.745]).

Neourethral-opening narrowing occurred in 14 out of the 48 eligible patients after grafting. It was managed through urethral dilation before proceeding to tubularization. Although it was successfully managed through a single or two instances of urethral dilation in 7 patients, only 4 out of the 7 cases (57.1%) demonstrated a successful outcome. The remaining 7 patients underwent periodic urethral dilation until tubularization. During tubularization, Heineke-Mikulicz-strictureplasty was required for 5 out of the 7 patients. Among them, success was observed in 3 out of the 5 patients (60%). Of the 2 patients who did not undergo Heineke-Mikulicz-strictureplasty, one achieved success, while the other did not.

In this context, we analyzed factors influencing the occurrence of neourethral-opening narrowing after grafting in the entire cohort (n = 67) (Table 3S in supplementary material). On multivariate logistic-regression analysis, the presence of hypertension (adjusted odds ratio [95% confidence-interval]: 7.799 [1.909–31.856]), longer stricture length (adjusted odds ratio [95% confidence-interval]: 1.376 [1.066–1.777]), and a history of prior DVIU (adjusted odds ratio [95% confidence-interval]: 5.871 [1.390–24.800]) were independent predictors of the occurrence of neourethral-opening narrowing.

## Discussion

A recent study showed a zero rate of re-grafting and an early success rate of 100.0% at 4-months after staged urethroplasty using thigh split-thickness skin graft (STSG) or BMG [3]. However, the long-term success rate of the study was only 53.0% in patients with a follow-up duration of > 1 year after staged urethroplasty using STSG [3]. Thus, STSG has a problem with durability. Meanwhile, despite the popular use of BMG in staged urethroplasties, its contracture rates were reported to be 20.0% to 39.0% after grafting, leading to multiple graft revision surgeries and high patient disappointment [16, 17]. These high contracture rates appear to be attributed to several factors, including exposure of BMG to dry air [16]. In this context, we took note of FTSG from the hairless groin area as a promising graft material for staged urethroplasty due to its advantages.

In this study, we aimed to report the surgical outcomes of staged urethroplasty using groin-FTSG, and to determine the factors influencing treatment outcomes. The major findings of our study can be summarized as follows: (1) Staged urethroplasty using hairless groin-FTSG showed acceptable success rate and low morbidity. (2) The rate of re-grafting was very low after the first-stage operation. (3) The presence of neourethral-opening narrowing after grafting was the only factor leading to non-success after tubularization. (4) In addition, the presence of hypertension, longer stricture length, and a history of prior DVIU were independent predictors of the occurrence of neourethral-opening narrowing after grafting.

In our study, the success rate of staged urethroplasty using groin-FTSG was 81.3%. Moreover, one out of the nine patients in the non-successful group had a satisfactory outcome after a single instance of urethral dilation. Although it may be difficult to directly compare study outcomes due to differences in the clinical characteristics of patients, including stricture length and definitions of success, our success rate is comparable to those of staged urethroplasty using other sources of graft materials, such as BMG (53.0–87.5%) [3, 12, 18–20]. In addition, our data showed a very low rate of re-grafting (4.5%) due to graft loss after the first-stage operation using hairless groin-FTSG. This falls within the range of the re-grafting rates (0.0–20.0%) reported in previous studies using other sources of graft materials [3, 12, 19]. In this context, our study extends the current state of graft materials for staged urethroplasty by, for the first time, showing the favorable outcomes of staged urethroplasty using groin-FTSG.

An important finding of our study is that the presence of neourethral-opening narrowing after grafting was the only factor influencing the unsuccessful outcome after tubularization. The incidence of neourethral-opening narrowing in our study (26.9%) falls within the range of the incidence reported in previous studies (3.3–29.8%) using BMG or post-auricular FTSG [2, 11, 21]. Although the first-stage operation is performed perfectly based on a principle, neourethral-opening narrowing due to annular contracture appears to develop in some patients after grafting. Andrich et al. reported that the re-stricture rate after tubularization was reduced through re-grafting in the patients with neourethral-opening narrowing, which had developed after grafting using BMG or post-auricular FTSG [11]. A recent study reported the surgical outcomes of staged urethroplasty using triangular extension to avoid neourethral-opening narrowing [21]. In that study, strips of BMG extending beside the stoma were placed over both the distal and proximal ends at opposite sides [21]. There was no difference in the incidence of neourethral-opening narrowing and the successful outcome between the triangular-extension group and conventional staged urethroplasty group (30.4% vs. 29.2%, and 95.0% vs. 95.0%, respectively) [21]. However, the triangular-extension group showed zero rate of re-grafting due to neourethral-opening narrowing, while the conventional urethroplasty group had a re-grafting rate of 20.8% [21]. In accordance with this, our study showed that there was no case in which additional grafting due to neourethral-opening narrowing after the first-stage operation was needed. In our study, successful outcomes were observed in only 8 out of the 14 cases in which neourethral-opening narrowing occurred after grafting. Furthermore, two out of the 6 patients who had unsuccessful outcomes underwent repeat staged urethroplasty but failed to achieve success. Taken together, the presence of neourethral-opening narrowing after grafting is an ominous sign of unsuccessful outcomes after tubularization.

We analyzed the factors influencing the occurrence of neourethral-opening narrowing. To our knowledge, our study is the first report showing that the presence of hypertension, longer stricture length, and a history of prior DVIU were associated with the occurrence of neourethral-opening narrowing. First, a longer skin graft is required for a lengthier stricture segment, placing a greater burden on vascularization during the graft take, thereby leading to ischemic contracture. Second, the presence of hypertension may have an adverse impact on vascular perfusion in skin graft through microangiopathy or arterial stiffness during the graft take [22]. Lastly, patients with a history of DVIU have a higher probability of having more profound spongiofibrosis compared to those without. Thus, we should pay more careful attention to the prevention of neourethral-opening narrowing through additional procedures such as placing wider strips of skin grafts around the neourethral-opening or obtaining more sufficient margins of healthy urethra proximally and distally to the stricture tract in patients with the risk factors. Recently, we performed the dorsal-inlay grafting through sagittal urethrotomy on the dorsal side of a narrowed neourethral-opening as an additional procedure during tubularization in a patient with neourethral-opening narrowing. This resulted in a successful outcome, even though the follow-up period has been short-term so far. We believe that this can be a potential solution for patients in whom neourethral-opening narrowing occurs after grafting, although a longer follow-up period is needed.

There are several limitations in our study. First, our study is retrospective, and lacks a large cohort. Second, we did not compare the outcomes between groin-FTSG and other sources of graft materials. Third, the number of patients with non-successful outcome or neo-urethral opening narrowing was small. Overfitting a regression model could occur because cases of the outcomes of interest were small. Lastly, the heterogeneity of surgical technique over the long time period can be a limitation of this study. Future-studies with large cohorts are needed to validate our findings. Nevertheless, our study carries clinical significance, in that staged urethroplasty using groin-FTSG can be a useful therapeutic option for complex anterior urethral strictures.

## Conclusion

Our data indicate that staged urethroplasty using groin-FTSG is well worth considering as a useful therapeutic option for complex anterior urethral strictures, with an acceptable success rate and low morbidity. The absence of neourethral-opening narrowing after grafting was associated with success after tubularization.

## Supplementary Information

Below is the link to the electronic supplementary material.

Supplementary file1 (PDF 288 KB)

## Data Availability

All inclusion data are available upon contact with authors.
