# Supplementary material for: Staged urethroplasty with groin full-thickness skin graft for managing complex anterior urethral strictures: surgical outcomes and predictive factors
Source: World J Urol. 2024 May 22;42(1):342. doi: 10.1007/s00345-024-05049-3 (PMC11111480; doi:10.1007/s00345-024-05049-3)
Supplement: Supplementary file 1 — Supplementary file1 (PDF 288 KB) [file 345_2024_5049_MOESM1_ESM.pdf]

Table 1S. Baseline characteristics and Perioperative data on the first-stage operation in all patients (n = 67)

**Variables**	**Values: Median (range) or n (%)**
**Baseline characteristics**	
Age, yr	64 (18 – 82)
Body mass index, kg/m^2	25.0 (16.0 – 32.0)
Diabetes mellitus	15 (22.4%)
Hypertension	22 (32.8%)
History of urethritis	3 (4.5%)
History of urinary tract infection	12 (17.9%)
Smoking (non-smoker / ex-smoker / current smoker)	52 (77.6%) / 2 (3.0%) / 13 (19.4%)
Stricture etiology (failed hypospadias repair / iatrogenic / idiopathic / traumatic)	13 / 38 / 12 / 4
Stricture location (penile / penobulbar / bulbar / panurethral)	33 / 28 / 1 / 5
Stricture length, cm	5.5 (1.0 – 12.0)
Type of prior interventions (dilation / DVIU / urethroplasty / urethral stent)	34 / 25 / 19 / 6
Presence of suprapubic catheter	26 (38.8%)
Pyuria / Bacteriuria	33 (49.3%) / 17 (25.4%)
Uroflowmetry	
- Maximum flow rate, mL/sec	3.0 (0 – 13.0)
- Post-void residual urine volume, mL	30.0 (0.0 – 361.0)
**Perioperative data on the first-stage operation**	

FTSG length / width, cm	7.0 (2.0 – 13.0) / 3.0 (2.0 – 5.0)
Urethrotomy including the urethral meatus	27 (40.3%)
Preservation of urethral plate	62 (92.5%)
Concomitant urethral surgery for bulbar urethral stricture (EPA or Substitution urethroplasty using BMG)	3 (4.5%) / 7 (10.4%)
Operation time, min	180 (75 – 300)
Duration of catheterization, day	16.0 (7.0 – 29.0)
Hospital stay, day	8.0 (5.0 – 19.0)
Complications at the recipient site [Clavien-Dindo classification]	
- narrowing of the neourethral opening	18 (26.9%) [IIIa in 18]
- partial loss of graft	8 (11.9%) [IIIb in 3 and II in 5]
- wound dehiscence	3 (4.5%)
Complications at the donor site [Clavien-Dindo classification]	
- wound dehiscence	3 (4.5%) [I in 3]
Re-grafting	3 (4.5%)
Reasons for re-grafting	
- loss of graft	3 (4.5%)
Proceed to tubularization (the second-stage)	59 (88.1%)
Time to tubularization after grafting, mo	5.1 (4.0 – 32.0)

DVIU: direct vision internal urethrotomy, FTSG: full-thickness skin graft, EPA: excision and primary anastomosis. BMG: buccal

mucosa graft

Table 2S. Comparison in baseline characteristics and the perioperative data on the first-stage operation between the patients with successful outcomes and those without

**Variables**	**Non-successful group (n = 9)**	**Successful group (n = 39)**	**p-value**
Values: Median (range) or n (%)			
**<u>Baseline characteristics</u>**			
Age, yr	59 (33 – 80)	60 (18 – 82)	0.716
Body mass index, kg/m^2	25.0 (20.0 – 27.0)	24.0 (19.0 – 32.0)	0.897
Diabetes mellitus	3 (33.3%)	10 (25.6%)	0.687
**Hypertension**	6 (66.7%)	9 (23.1%)	**0.018**
History of urethritis	0 (0.0%)	2 (5.1%)	0.661
History of urinary tract infection	1 (11.1%)	9 (23.1%)	0.861
Smoking (non-smoker / ex-smoker / current smoker)	8 (88.9%)/0 (0.0%)/1 (11.1%)	29 (74.4%)/2 (5.1%)/8 (20.5%)	0.779
Indications for staged urethroplasty			0.341
- previous failed hypospadias surgery	0 (0.0%)	9 (23.1%)	
- previous failed urethroplasty	4 (44.4%)	12 (30.8%)	
- very poor tissue quality of urethra related to multiple previous dilation or DVIU	5 (55.6%)	18 (46.2%)	
Stricture etiology (failed hypospadias repair / iatrogenic / idiopathic / traumatic)	0 (0.0%) / 7 (77.8%) / 2 (22.2%) / 0 (0.0%)	10 (25.6%) / 20 (51.3%) / 6 (15.4%) / 3 (7.7%)	0.290

Stricture location (penile / penobulbar / panurethral)	3 (33.3%)/6 (66.7%)/0 (0.0%)	19 (48.7%)/18 (46.2%)/2 (5.1%)	0.649
Stricture length, cm	6.0 (4.5 – 8.5)	5.5 (1.0 – 12.0)	0.390
Type of prior interventions			
- dilation	6 (66.7%)	24 (61.5%)	1.000
- DVIU	6 (66.7%)	19 (48.7%)	0.466
- urethroplasty	3 (33.3%)	12 (30.8%)	1.000
- urethral stent	2 (22.2%)	3 (7.7%)	0.231
Presence of suprapubic catheter	4 (44.4%)	16 (41.0%)	1.000
Pyuria / Bacteriuria	6 (66.7%) / 2 (25.0%)	18 (46.2%) / 10 (26.3%)	0.461
**Perioperative data on the first-stage operation**			
FTSG length / width, cm	7.0 (6.0 – 10.0) / 3.0 (2.0 – 3.5)	7.0 (2.0 – 11.0) / 3.0 (2.0 – 5.0)	0.585
Urethrotomy including the urethral meatus	3 (33.3%)	15 (38.5%)	1.000
Preservation of urethral plate	9 (100.0%)	35 (89.7%)	1.000
Concomitant urethral surgery for bulbar stricture (EPA or Substitution urethroplasty using BMG)	2 (22.2%)	6 (15.4%)	1.000
Operation time, min	180.0 (150.0 – 230.0)	185.0 (75.0 – 300.0)	0.716
Duration of catheterization, day	15.0 (10.0 – 23.0)	16.0 (7.0 – 29.0)	0.815
Hospital stay, day	9.0 (7.0 – 19.0)	8.0 (5.0 – 17.0)	0.141
Location of proximal neourethral opening			0.478
- Penile	3 (33.3%)	19 (48.7%)	

- Bulbar	6 (66.7%)	20 (51.3%)	
Complications at the recipient site			
- **narrowing of the neourethral opening**	6 (66.7%)	8 (20.5%)	**0.012**
proximal	6	6	
distal	1	3	
- graft necrosis	1 (11.1%)	2 (5.1%)	0.472
- partial loss of graft	1 (11.1%)	5 (12.8%)	1.000
Management of neourethral opening narrowing			
- a single or two instances of urethral dilation	3	4	
- periodic urethral dilation	3	4	
- Heineke-Mikulicz strictureplasty	2	3	
Re-grafting	1 (11.1%)	1 (2.6%)	0.343
Reasons for re-grafting			
- loss of graft	2 (22.2%)	1 (2.6%)	0.086
Time to tubularization after grafting, mo	5.1 (4.2 – 7.9)	4.8 (4.0 – 19.1)	0.640

DVIU: direct vision internal urethrotomy, FTSG: full-thickness skin graft, EPA: excision and primary anastomosis. BMG: buccal mucosa graft

Table 3S. Comparison in baseline characteristics and the perioperative data on the first-stage operation between the patients with neourethral opening narrowing and those without

**Variables**	**patients with neourethral orifice narrowing (n = 18)**	**patients without neourethral orifice narrowing (n = 49)**	**p-value**
Values: Median (range) or n (%)			
**<u>Baseline characteristics</u>**			
**Age**, yr	72 (41 – 82)	59.0 (18 – 82)	**0.023**
Body mass index, kg/m^2	24.5 (20.0 – 29.0)	25.0 (18.0 – 32.0)	0.669
Diabetes mellitus	6 (33.3%)	9 (18.8%)	0.322
**Hypertension**	11 (61.1%)	11 (22.9%)	**0.007**
History of urethritis	0 (0.0%)	3 (6.3%)	0.556
History of urinary tract infection	1 (5.6%)	11 (22.9%)	0.212
Smoking (non-smoker / ex-smoker / current smoker)	16 (88.9%)/2 (11.1%)/0 (0.0%)	35 (72.9%)/0 (0.0%)/13 (27.1%)	0.052
Indications for staged urethroplasty			0.058
- previous failed hypospadias surgery	0 (0.0%)	9 (23.1%)	
- previous failed urethroplasty	7 (38.9%)	12 (30.8%)	
- very poor tissue quality of urethra related to multiple previous dilation or DVIU	11 (61.1%)	24 (50.0%)	
Stricture etiology (failed hypospadias repair /	0 (0.0%) / 14 (77.8%)	13 (27.1%) / 24 (50.0%)	0.216

iatrogenic / idiopathic / traumatic)	/ 1 (5.6%) / 3 (16.7%)	/ 10 (20.8%) / 1 (2.1%)	
**Stricture location**	4 (22.2%) / 10 (55.6%)	28 (58.3%) / 18 (37.5%)	**0.008**
(penile / penobulbar / bulbar / panurethral)	/ 0 (0.0%) / 4 (22.2%)	/ 1 (2.1%) / 1 (2.1%)	
**Stricture length**, cm	6.5 (3.0 – 12.0)	5.0 (1.0 – 11.0)	**0.016**
Type of prior interventions			
- dilation	13 (72.2%)	25 (52.1%)	0.170
- **DVIU**	11 (61.1%)	16 (33.3%)	**0.049**
- urethroplasty	5 (27.8%)	13 (27.1%)	1.000
- urethral stent	2 (11.1%)	3 (6.3%)	0.608
Presence of suprapubic catheter	9 (50.0%)	17 (35.4%)	0.397
Pyuria / Bacteriuria	7 (38.9%) / 4 (22.2%)	25 (52.1%) / 12 (26.1%)	0.413
**Perioperative data on the first-stage operation**			
FTSG length, cm	8.33 (5.0 – 13.0)	6.48 (2.0 – 12.0)	0.004
FTSG width, cm	3.0 (2.0 – 4.0)	3.0 (2.0 – 5.0)	0.104
Urethrotomy including the urethral meatus	5 (27.8%)	21 (40.9%)	0.272
Preservation of urethral plate	18 (100.0%)	43 (89.6%)	0.312
Concomitant urethral surgery for bulbar stricture	3 (16.7%)	8 (16.7%)	0.556
(EPA or Substitution urethroplasty using BMG)			
Operation time, min	187.5 (140 – 245)	170.0 (75.0 – 300.0)	0.220
Duration of catheterization, day	18.56 (10.0 – 29.0)	15.33 (7.0 – 28.0)	0.815

Hospital stay, day	9.78 (7.0 – 19.0)	8.5 (5.0 – 17.0)	0.073
**Location of proximal neourethral opening**			**0.012**
- Penile	4 (22.2%)	29 (59.2%)	
- Bulbar	14 (77.8%)	20 (40.8%)	
Complications at the recipient site			
- graft necrosis	1 (5.6%)	5 (10.4%)	1.000
- partial loss of graft	2 (11.1%)	6 (12.5%)	1.000
Re-grafting	2 (11.1%)	2 (4.2%)	1.000
**Time to tubularization after grafting**, mo	6.5 (4.2 – 9.3)	4.8 (4.0 – 32.0)	**0.002**

DVIU: direct vision internal urethrotomy, FTSG: full-thickness skin graft, EPA: excision and primary anastomosis. BMG: buccal mucosa graft

Figure 2S. Follow-up data on uroflowmetry after the second-stage operation (tubularization) in eligible patients (n = 48).

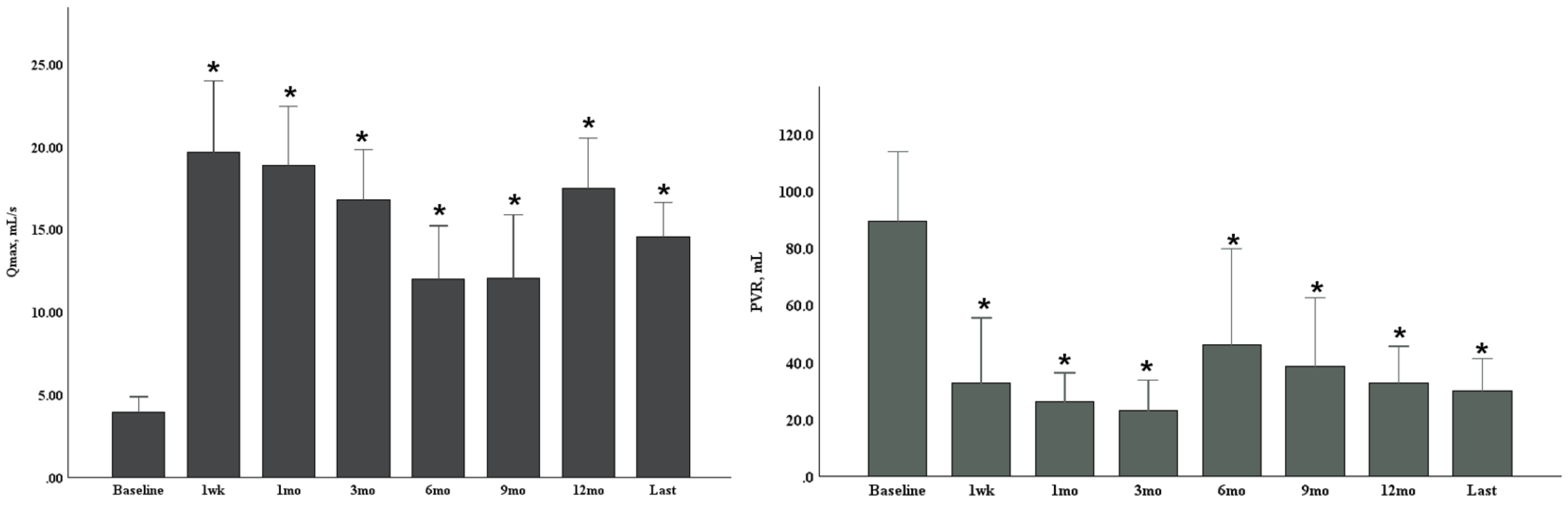

Qmax = Maximum urine flow rate, PVR = post-void residual urine volume, last = the last follow-up visit
